# The potential impact of a recent measles epidemic on COVID-19 in Samoa

**DOI:** 10.1186/s12879-020-05469-7

**Published:** 2020-10-07

**Authors:** Chandini Raina MacIntyre, Valentina Costantino, David J. Heslop

**Affiliations:** 1 The Biosecurity Program, The Kirby Institute, UNSW Medicine, The University of New South Wales, Sydney, Australia; 2 School of Public Health and Community Medicine, The University of New South Wales, Sydney, Australia

**Keywords:** Immune paresis, Measles immunity amnesia, Coronavirus

## Abstract

**Background:**

The pandemic of COVID-19 has occurred close on the heels of a global resurgence of measles. In 2019, an unprecedented epidemic of measles affected Samoa, requiring a state of emergency to be declared. Measles causes an immune amnesia which can persist for over 2 years after acute infection and increases the risk of a range of other infections.

**Methods:**

We modelled the potential impact of measles-induced immune amnesia on a COVID-19 epidemic in Samoa using data on measles incidence in 2018–2019, population data and a hypothetical COVID-19 epidemic.

**Results:**

The young population structure and contact matrix in Samoa results in the most transmission occurring in young people < 20 years old. The highest rate of death is the 60+ years old, but a smaller peak in death may occur in younger people, with more than 15% of total deaths in the age group under 20 years old. Measles induced immune amnesia could increase the total number of cases by 8% and deaths by more than 2%.

**Conclusions:**

Samoa, which had large measles epidemics in 2019–2020 should focus on rapidly achieving high rates of measles vaccination and enhanced surveillance for COVID-19, as the impact may be more severe due to measles-induced immune paresis. This applies to other severely measles-affected countries in the Pacific, Europe and elsewhere.

## Strengths and limitations of this study

- This modelling study include heterogeneity of the Samoa population age structure and age specific contacts
- The model includes the effect of non-pharmaceutical interventions in containing the outbreak, like contacts tracing, quarantine, and isolation
- The force of infection is modelled to be heterogeneous over the infectious period
- The uncertain of the parameters used, especially regarding the infectiousness and proportion of asymptomatic infections in the age group < 10 years old is one of the limitations of this study.

## Background

The pandemic of COVID-19 has occurred close on the heels of a global resurgence of measles. Europe, Asia and the Pacific have experienced unprecedented measles epidemics in 2019 [1]. Other than direct viral morbidity and mortality, measles causes an immune amnesia which can persist for over 2 years after acute infection and increases the risk of a range of other infections [2–6]. The mechanism is thought to be through loss of memory B cells and plasma cells which produce antibodies to a range of infections [7]. The measles virus is also shown to directly infect memory CD4^+^ and CD8^+^ T cells as well as naïve T cells [8]. A study in Switzerland showed that measles immune amnesia increases the risk of a wide range of infections, with a relative risk of 3.47 for other infections following measles [2]. This risk may be higher in low income countries where malnutrition is an exacerbating factor. The corollary is that measles vaccination can protect against non-measles infections, presumably by preventing immune dysfunction [9]. In the Democratic Republic of Congo, measles vaccine protected against fever, cough and diarrhoea, presumably of non-measles causes [10].

In 2019, an unprecedented epidemic of measles affected Samoa. A state of emergency was declared on November 15th and lifted on December 2019, with a peak of the epidemic around November 26th and up to 3% of the population infected (5707 cases of 199,955 people) with 87 deaths [11]. The highest rates of infection were in the age groups 6–11 months, 0–5 months and 1–4 years, with 87% of deaths in children under 5 years. This translates to 8% of the population under 15 years being infected with measles within the last 6 months [12]. Over 35% of the Samoan population (70,694) is aged < 15 years, and only 5.2% is over 65 years old.

Based on available data, COVID-19 causes more severe illness in older people, and children may be asymptomatic, especially under the age of 10 [13, 14]. However, in a case series of over 2000 children with COVID-19, only 50% had mild infection, 6% had critical illness and 1 child aged 14 years died [15]. In another case series, a child aged 10 months died of COVID-19 [16].

We previously exercised a hypothetical pandemic in the Pacific, with involvement of multiple Pacific island nations, and highlighted the unique challenges of the region [17]. These include multiple islands, small populations, natural disasters, weak health systems and poor disease surveillance infrastructure [18]. The impact of measles induced immune amnesia on the manifestation of COVID-19 is unknown and has not been considered, despite a global resurgence of measles in 2019. COVID-19 in Samoa could have a severe impact and a disproportionate increase in morbidity and mortality due the medium-term immune dysregulation caused by the recent measles epidemic.

The aim of this study is to estimate the age-specific morbidity and mortality impact of COVID-19 in Samoa, accounting for the potential impact of measles-induced immune amnesia.

## Methods

We used data from the WHO situation reports of the measles epidemic in Samoa [11, 19, 20], and an assumption that immune amnesia would be present in children infected from November 2019 onward for at least 12 months and up to 36 months [2], to model the morbidity and mortality impact of a COVID-19 epidemic in the country. We used the Samoa population for 2020 [21], which we divided by age groups following the age distribution in 2016 [22].

The entire population was considered susceptible to COVID-19 and the hypothetical epidemic starts at t = 1 with 6 latently infected people. After symptom onset, we assumed that only 80% of symptomatic infected people get effectively isolated after 5 days [23], and isolation is assumed to stop further transmission. However asymptomatic cases, assumed to be 35% of all infections [24–26] will not be isolated because they will not be detected due to weak health systems and limited testing capacity in Samoa. Detection of asymptomatic cases requires substantially expanded testing capacity and testing of all close contacts of a case, regardless of symptoms – this is unlikely in small Pacific islands, which may struggle to even test all symptomatic cases. We considered the latent or asymptomatic period to be infectious during the last 2 days before symptoms onset, causing 44% of the total transmissions [27], and assumed the highest viral load just prior to and just after symptoms [26, 28–31]. Regarding the symptomatic period, we modelled the first day as the most infective, following by a decreasing in the transmissions potential for the following 6 days [27], making the infectious symptomatic period 7 days long [30]. Accounting for weak health systems and limited human resource capacity, we assumed that only 60% of known contacts of an infected person will be quarantined for 14 days, and if they are latent and become symptomatic, they will take 2 days to be isolated, however we assumed that only 80% of symptomatic people will be effectively isolated without further transmissions.

We used an age-specific deterministic model which simulates the epidemic in Samoa running for 400 days. The model moves people between 13 mutually exclusive compartments: Susceptible (S), Latent not infectious (E), Latent infectious undiagnosed (Eu) and diagnosed (Et), first symptomatic day for undiagnosed (I1) and diagnosed (I2), following 6 days of symptomatic for undiagnosed (I11) and diagnosed (I22), Asymptomatic infectious stages (A1 and A2), Isolated (Q), Recovered (R) and Dead (D). Each of those compartments is divided in 16 age stratified groups each of 5 years duration, ranging from 0 to 74 years old plus an additional age group of 75+ years, as available from the population data for Samoa. The force of infection that moves people from the susceptible compartment to the latent one is age-specific, based on average age-specific contact rates for Samoa [32], the R0 and the proportion of infected people over the entire population.

We considered transmission to be the same in adults and children based on viral shedding data that suggest this [33], where children often present very milder symptom or none [34–36]. Studies showed children under 5 years have a higher viral load in the respiratory tract than adults or older children [37–39]. Another study showed that COVID-19 attack rates in families were higher when the primary case was a child [40]. In the United States, 9.8% of all cases of COVID-19 were children [41]. Given the role of children in transmission remains an area of uncertainty, we conservatively assumed equal transmission potential from children and adults. Failure to seroconvert has also been documented in people with asymptomatic infection [42], so lack of seropositivity may not equate to lack of infection, as infection may be asymptomatic or mild may not result in seroconversion in children < 10 years [43].

To include the effect of measles on the immune system of COVID-19 infected people, we used the total number of reported cases of measles in 2019 in Samoa, 5707 [11] and, due to the lack of detailed individual age data for those cases, we assumed that 90% were in the first age-group 0–4 years, 5% in the 5–9 age group and 5% in the 10–14 age group, based on the data showing that 87% of all deaths were reported in the age-group younger than 5 [11]. Once we distributed cases in those three age groups, we calculated the proportion of people that had measles with respect to the age group size.

Based on a three-year observational study, immunological amnesia after measles can increase the risk of infectious disease hospitalizations by 3.47 times [2]. For the people who had been infected with measles in the preceding 12 months, we than assumed that they are 3.47 times more likely to get infected with COVID-19. However, we averaged the susceptibility of each age-group, weighting it between the proportion that had measles against those that did not, and overall calculated that the first three age groups, 0–4, 5–9 and 10–14 are 1.44, 1.03 and 1.03 times more likely to get infected respectively. Model parameters are shown in Table 1, while the model equations are described in the supplementary material.

**Table 1:** Parameters used in the model

Parameter	Value	Source
Basic reproduction number	2.5	[26] {Li, 2020 #5}
Infectious period	9 days of which 2 in latency and 7 symptomatic	[27]
Increased susceptibility to COVID-19 due to measles	1.44 for 0–4 years old 1.03 for 5–9 years old 1.03 for 10–14 years old	Calculated
Time to isolation once symptomatic	5 days	[44]
Effectiveness of home quarantine in latency period	50% reduction in the R0	[45]
Effectiveness of isolation	100% (no transmissions)	
Duration of home quarantine	14 days	WHO recommendation [46]
Duration of isolation	14 days	
Proportion of asymptomatic or very mild infectious	35%	[24–26]
Proportion of contacts identified for home quarantine	60%	[23]
Proportion of symptomatic people that get isolated after 5 days	80%	[23]
Age-specific case fatality rate (%) for the 16 age groups	0.1, 0.1, 0.2, 0.2, 0.2, 0.2, 0.2, 0.2, 0.4, 0.4, 1.3, 1.3, 3.6, 3.6, 8, 14.8	[47]

### Patient and public involvement

No patients were involved in the design of this study, development of research questions and outcome measures.

### Data sharing

No additional data available.

## Results

**Fig. 1:**
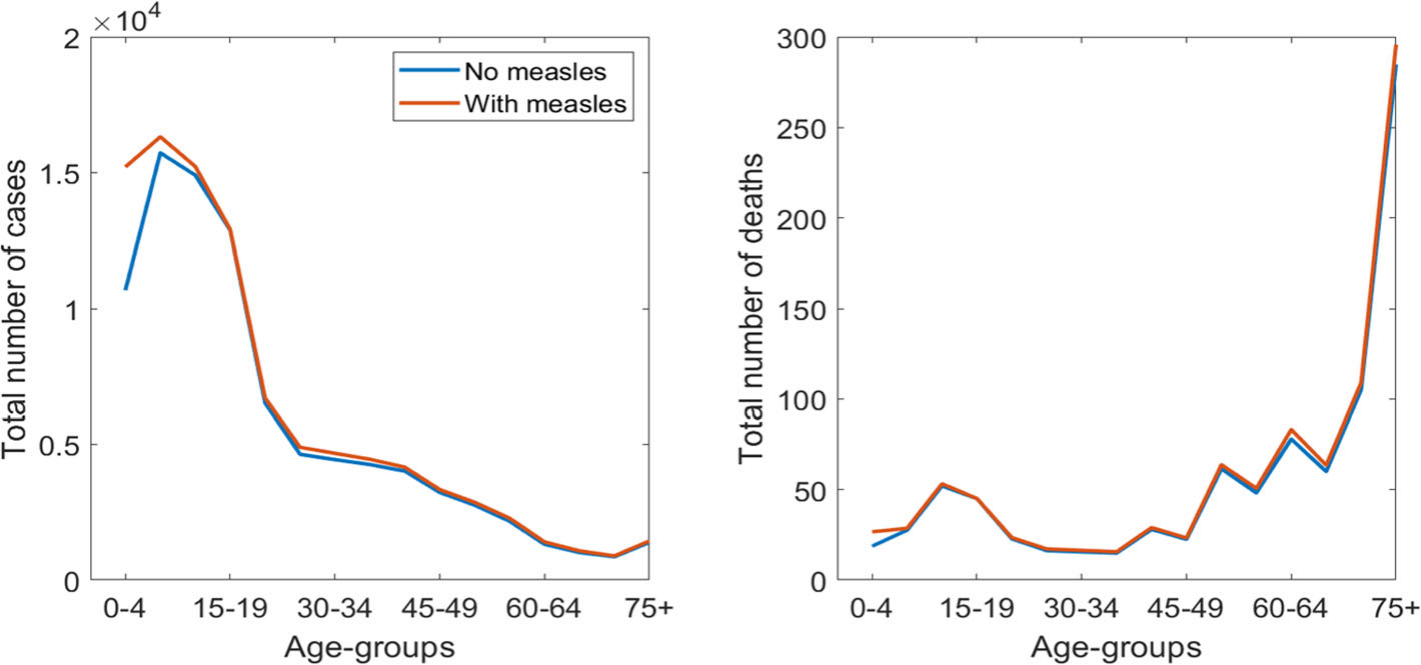
Cumulative age-specific cases (left) and deaths (right) with and without measles immunity paresis effect

**Fig. 2:**
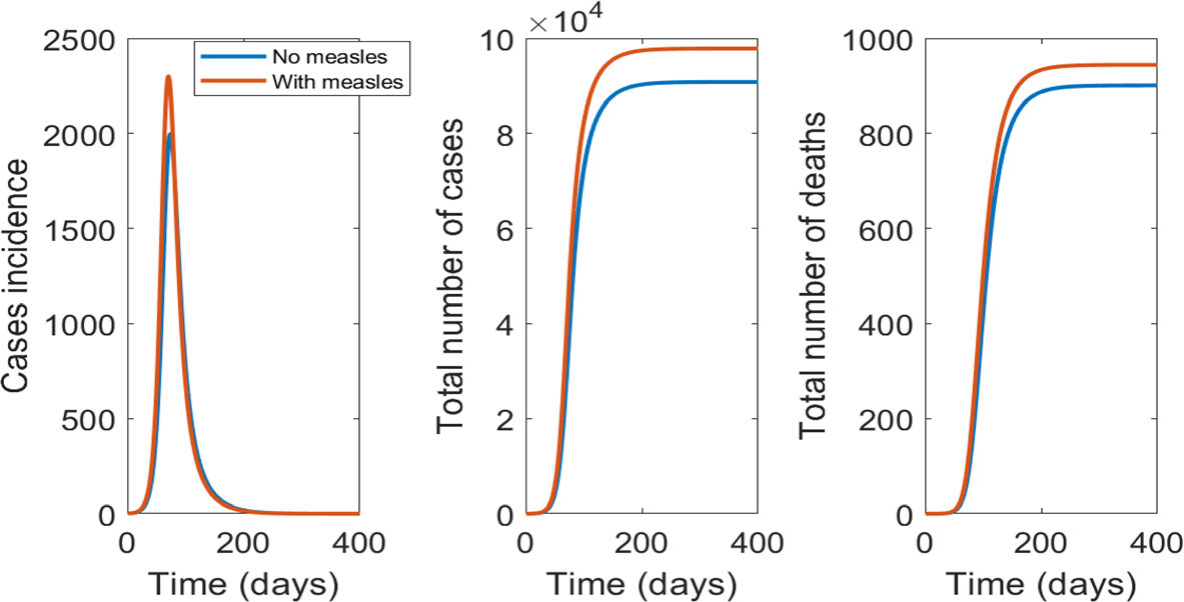
From left to right: cases incidence, cumulative cases and deaths with and without measles immunosuppression

In Fig. 1 we show the age distribution of infections on the left and deaths on the right by the end of the epidemic. With or without measles effect, about 60% of infections are in the age group younger than 20 years old. Without the measles effect, 11.76, 17.33, 16.42 and 14.18%of the total cases are in the age group 0–4, 5–9, 10–14 and 15–19 respectively, with deaths in the under 20 years old accounting for more than 15% of the total deaths. Most deaths occur in older age groups. With measles immune paresis, the proportion of cases in the age group 0–4 represents 15.55% of the total cases and in the following two age-groups it is 16.69 and 15.57% of the total number of cases, respectively. Measles immune paresis increases the number of cases by 42% in the first age group (from 10,680 cases to 15,213), almost 4% in the second age group and just over 2% in the third age group; while deaths in the age group 0–4 see a 59% increase (from 17 to 27). This corresponds to an increase of almost 8% (from 90,784 to 97,824) in the total number of cases and an increase of 2.3% (from 901 to 944) in the number of total deaths associated with measles immune paresis (Fig. 2).

## Discussion

The population of Samoa has experienced a recent, severe measles epidemic, and as of the end of June 2020 not yet reported any cases of COVID-19. The impact of measles immune paresis is well documented [2, 4, 7, 8, 48], but has not been considered as a factor which may influence COVID-19 epidemiology to date. In the first study considering this, we show a potential increase of morbidity and mortality following the spread of COVID-19 in Samoa. The immune effects of measles would be most prevalent in the most measles-affected age group in Samoa, children, resulting in potential increases in morbidity and mortality in this age group. Samoa has a young age structure and intense contact between younger age groups driving the observed COVID-19 transmission in the model, and this is compounded by the high attack rate of measles in children in 2019. A large proportion of infection in children is asymptomatic or mild, with the probability of symptomatic disease increasing with age. However, available data show that severe morbidity and mortality can occur in children [15]. Deaths due to COVID-19 have been documented in infants [16]. The effect of the severe measles epidemic may result in higher severity of disease in children in Samoa. It is also now clear based on viral shedding data that children play an important role in transmission [33], and may even have a higher viral load in the respiratory tract than adults [37]. A household contact study found that COVID-19 attack rates in families were higher when the primary case was a child [40].

The age specific mixing rates in most countries show a peak in social mixing in young people and children, corresponding to increased infection transmission for most infections in these age groups [32, 49]. Therefore, if COVID-19 is introduced into Samoa, children and young people may drive transmission, which could place older people at risk due to extended family structures and the importance of community gatherings in Pacific islands. There are few data on COVID-19 in the Pacific, but one study reports poorer outcomes in Pacific Islanders in Hawaii, attributed to lifestyle diseases and comorbidities such as diabetes [50].

For these reasons, prevention and control of COVID-19 in Samoa is critical. Disease control measures such as travel bans, case isolation, contact tracing, quarantine, face masks and social distancing are the only available interventions for COVID-19 at this time, and Samoa has effectively closed its international borders [23]. For a young population like Samoa, school closure would be an effective disease control strategy for both measles and COVID-19 [32]. Social distancing may be particularly difficult in Pacific cultures, where large gatherings and extended families are the norm, so face mask use should be considered. Ensuring high levels of vaccination against measles is also critical to reduce the potential additional risk of measles immune paresis.

A study of 2143 paediatric cases in China found only 50% had mild disease, 30% had moderate disease, 6% were critically ill and one child aged 14 years died [15]. In another study, of 171 cases of COVID-19 in China, the median age was 6.7 years, three required ventilation and an infant aged 10 months died [16]. This accumulating evidence, including recent association with Kawasaki disease, shows that severe illness and death is possible in children. The occurrence of post-COVID-19 Multisystem Inflammatory Syndrome, or Kawasaki Syndrome, in children with a median age of 8 years, is associated with high morbidity and mortality [51], but the impact of this syndrome was not considered in the model. It is unknown whether measles immune paresis would reduce or increase the risk of Kawasaki disease after COVID-19 infection, but research is warranted in this area.

## Conclusions

Whilst children may be more likely to be asymptomatically infected, reflected by a low proportion of all cases being children [47] a background of a compromised immune system induced by recent measles infection may change the age-specific epidemiology in countries with recent, widespread measles transmission. These considerations may also affect other countries which have had recent measles epidemics in the Pacific, in Europe and elsewhere. These countries must strongly focus on achieving high levels of measles vaccination as part of the COVID-19 control strategy.

## Supplementary information

**Additional file 1.**

## Data Availability

For measles cases in Samoa, this study uses open access published data available from WHO reports at https://www.who.int/immunization/monitoring_surveillance/burden/vpd/surveillance_type/active/measles_monthlydata/en/, while to inform the model for the spread of COVID-19 in Samoa we used previously estimated parameters from literature. All data used are open access.
