# Supplementary material for: The potential impact of a recent measles epidemic on COVID-19 in Samoa
Source: BMC Infect Dis. 2020 Oct 7;20:735. doi: 10.1186/s12879-020-05469-7 (PMC7539273; doi:10.1186/s12879-020-05469-7)
Supplement: Supplementary file 1 — Additional file 1. [file 12879_2020_5469_MOESM1_ESM.docx]

**COVID-19 Supplementary materials**

**Mathematical model**

The model presented here is a deterministic compartmental disease transmission model built using Matlab 2019. It is an expended SEIR system of ordinary differential equations, where the population (the initial susceptible) is divided in 16 age groups (i=1,…,16). The differential equations move the population through diseases epidemiological stages, as susceptible (S),latent not infectious yet (E) latent infectious undiagnosed (E^u^), infectious (I) and recovered (R) or death (D), and public health response stages, as latent infectious diagnosed (E^t^) or isolated (Q), with separate infectious states for asymptomatic people (A).

We used the following differential equations to simulate the epidemic spread of COVID-19 in Samoa.

$${dS}_{i}/d=-m_{i}*\lambda*S_{i}$$

$$dE_{i}/d=m_{i}*\lambda*S_{i}-E_{i}/d_{0}$$

$${dE}_{i}^{t}/d=\rho*E_{i}/d_{0}-E_{i}^{t}/d_{1}$$

$${dE}_{i}^{u}/d=(1-\rho)*E_{i}/d_{0}-E_{i}^{u}/d_{1}$$

$${dI}_{i}^{1}/d={(1-g)*E}_{i}^{t}/d_{1}-I_{i}^{1}/d$$

$${dI}_{i}^{2}/d={(1-g)*E}_{i}^{u}/d_{1}-I_{i}^{2}/d$$

$${dA}_{i}^{1}/d={g*E}_{i}^{t}/d_{1}+{g*E}_{i}^{u}/d_{1}-A_{i}^{1}/d$$

$${dI}_{i}^{11}/d=I_{i}^{1}/d-I_{i}^{11}/d$$

$${dI}_{i}^{22}/d=I_{i}^{2}/d-\theta*I_{i}^{22}/d_{4}-(1-\theta) *I_{i}^{22}/d_{6}$$

$${dA}_{i}^{2}/d=A_{i}^{1}/d-A_{i}^{2}/d_{6}$$

$${dQ}_{i}/d=I_{i}^{11}/d+\theta*I_{i}^{22}/d_{4}-\mu_{i}*Q_{i}/d_{o1}-(1-\mu_{i})*Q_{i}/q_{2}-\mu_{i}*Q_{i}/d_{5}$$

$${dR}_{i}/d=A_{i}^{2}/d_{6}+(1-\mu_{i})*(1-\theta) *I_{i}^{22}/d_{6}+g*I_{i}^{22}/d_{6}+(1-\mu_{i})*Q_{i}/q_{2}$$

$${dD}_{i}/d=\mu_{i}*(1-\theta) *I_{i}^{22}/d_{6}+\mu_{i}*Q_{i}/d_{5}$$

The force of infection is described as

$$\lambda_{i}=\sum_{j=1}^{18} \frac{\beta_{1}*c_{i,j}*E_{j}^{u}}{N}+\sum_{j=1}^{18} \frac{\beta_{2}*c_{i,j}*E_{j}^{t}}{N}+\sum_{j=1}^{18} \frac{\beta_{3}*c_{i,j}*{(I}_{j}^{1}+I_{j}^{2}+A_{i}^{1})}{N}+\sum_{j=1}^{18} \frac{\beta_{4}*c_{i,j}*{(I}_{j}^{11}+I_{j}^{22}+A_{i}^{2})}{N}$$

Where $\beta_{1}=\frac{0.44*R0}{d_{1}}$ for latent undiagnosed contacts, $\beta_{2}=\frac{\beta_{1}}{2}$ for latent diagnosed and home quarantined (50% reduction in R0),$\beta_{3}=0.36*R0$for the first day of symptoms and $\beta_{4}=\frac{0.2*R0}{d_{6}}$ for the following 6 days of symptoms$. c_{i,j}$ is the age-specific contact matrix adapted from (1) for Samoa, and *N* is the total population. The following table shows all the parameters used and their value.

Table S3: model parameters used and their values

| **Parameter** | **Symbol** | **Value** | **Source** |
| --- | --- | --- | --- |
| Basic reproduction number | R0 | 2.5 | (2) |
| Latent pre symptomatic period | $d_{0}+d_{1}$ | 3.2 not infectious +2 infectious =5.2 | (3) |
| Infectious period | $d_{1}+d+d_{6}$ | 2+1+6=9 days of which 2 presymptomatic, first day symptomatic with higher transmissions and following 6 days symptomatic with lower transmissions | (2,4–8) |
| Time to get isolated once symptomatic | $d+d_{4}$ | 1+4=5 days | (9) |
| Effectiveness of home quarantine | R0/2 | 50% reduction in the R0 | (10) |
| Duration of isolation | $q_{2}$ | 15 days |  |
| Time in isolation before dying | $d_{5}$ | 5 days |  |
| Increased susceptibility to COVID infection due to measles immune paresis effect on the first 3 age groups | $m_{i}$ | [1.44 1.03 1.03 1 1 1 1 1 1 1 1 1 1 1 1 1] | Calculated |
| Proportion of asymptomatic or very mild infectious | $g$ | 35% | (2,13,14) |
| Proportion of contacts identified for home quarantine | $\rho$ | 60% | (15) |
| Proportion of symptomatic people that get isolated after 5 days | $\theta$ | 80% | (15) |
| Age-specific case fatality rate (%) for the 18 age groups | $\mu_{i}$ | 0.1, 0.1, 0.2, 0.2, 0.2, 0.2, 0.2, 0.2, 0.4, 0.4, 1.3, 1.3, 3.6, 3.6, 8, 14.8 | (16) |

**References**

1. Prem K, Cook AR, Jit M. Projecting social contact matrices in 152 countries using contact surveys and demographic data. PLoS Comput Biol. 2017 Sep 1;13(9):e1005697.

2. COVID-19 Pandemic Planning Scenarios | CDC [Internet]. [cited 2020 Jun 29]. Available from: https://www.cdc.gov/coronavirus/2019-ncov/hcp/planning-scenarios.html

3. Li Q, Guan X, Wu P, Wang X, Zhou L, Tong Y, et al. Early transmission dynamics in Wuhan, China, of novel coronavirus–infected pneumonia. N Engl J Med. 2020;

4. Lin Y; Xie, B; et al CD. Asymptomatic Novel Coronavirus Pneumonia Patient Outside Wuhan: The Value of CT Images in the Course of the Disease. Clin Imaging. 2020;63:7–9.

5. Rothe M; Sothmann, P; et al CS. Transmission of 2019-nCoV Infection from an Asymptomatic Contact in Germany. N Engl J Med. January 30. 2020;

6. Zou L, Ruan F, Huang M, Liang L, Huang H, Hong Z, et al. SARS-CoV-2 Viral Load in Upper Respiratory Specimens of Infected Patients. N Engl J Med [Internet]. 2020; Available from: https://www.nejm.org/doi/full/10.1056/NEJMc2001737

7. Du Z, Xu X, Wu Y, Wang L, Cowling BJ, Meyers LA. Serial Interval of COVID-19 among Publicly Reported Confirmed Cases. Emerg Infect Dis [Internet]. 2020 Mar 19 [cited 2020 Mar 24];26(6). Available from: http://www.ncbi.nlm.nih.gov/pubmed/32191173

8. He X, Lau EHY, Wu P, Deng X, Wang J, Hao X, et al. Temporal dynamics in viral shedding and transmissibility of COVID-19. Nat Med. 2020 May 1;26(5):672–5.

9. Wang D, Hu B, Hu C, Zhu F, Liu X, Zhang J, et al. Clinical Characteristics of 138 Hospitalized Patients With 2019 Novel Coronavirus–Infected Pneumonia in Wuhan, China. JAMA [Internet]. 2020; Available from: https://doi.org/10.1001/jama.2020.1585

10. Zhang S, Diao MY, Yu W, Pei L, Lin Z, Chen D. Estimation of the reproductive number of novel coronavirus (COVID-19) and the probable outbreak size on the Diamond Princess cruise ship: A data-driven analysis. Int J Infect Dis. 2020 Apr 1;93:201–4.

11. Gudbjartsson DF, Helgason A, Jonsson H, Magnusson OT, Melsted P, Norddahl GL, et al. Spread of SARS-CoV-2 in the Icelandic Population. N Engl J Med [Internet]. 2020 Jun 11 [cited 2020 Jun 30];382(24):2302–15. Available from: http://www.nejm.org/doi/10.1056/NEJMoa2006100

12. Behrens JD; Heininger U LC. The Susceptibility to Other Infectious Diseases Following Measles During a Three Year observation Period in Switzerland. Pediatr Infect Dis J. January 10. 2020;

13. Mizumoto K, Chowell G. Transmission potential of the novel coronavirus (COVID-19) onboard the diamond Princess Cruises Ship, 2020. Infect Dis Model. 2020 Jan 1;5:264–70.

14. Estimating the asymptomatic ratio of 2019… | Oxford Martin School [Internet]. [cited 2020 Mar 24]. Available from: https://www.oxfordmartin.ox.ac.uk/publications/estimating-the-asymptomatic-ratio-of-2019-novel-coronavirus-onboard-the-princess-cruises-ship/

15. MacIntyre Valentina; Kunasekaram, Mohana Priya CRC. Health system capacity in Sydney,Australia in the event of a biological attack with smallpox. PLoS One. June 14 20. 2019;14(6).

16. Team TNCPERE. Vital Surveillances: The Epidemiological Characteristics of an Outbreak of 2019 Novel Coronavirus Diseases (COVID-19) — China, 2020. China CDC Wkly. 2020;2(8):113–22.
